# ^1^H, ^13^C, ^15^N chemical shift assignments of SHP2 SH2 domains in complex with PD-1 immune-tyrosine motifs

**DOI:** 10.1007/s12104-020-09941-y

**Published:** 2020-04-01

**Authors:** Michelangelo Marasco, John P. Kirkpatrick, Teresa Carlomagno

**Affiliations:** 1 Center for Biomolecular Drug Design and Institute of Organic Chemistry, Leibniz University Hannover, Schneiderberg 38, 30167 Hannover, Germany; 2 Helmholtz Center for Infection Research, Group of NMR-Based Structural Chemistry, Inhoffenstrasse 7, 38124 Braunschweig, Germany

**Keywords:** PD-1, SHP2, Immunotherapy, SH2 domains

## Abstract

Inhibition of immune checkpoint receptor Programmed Death-1 (PD-1) via monoclonal antibodies is an established anticancer immunotherapeutic approach. This treatment has been largely successful; however, its high cost demands equally effective, more affordable alternatives. To date, the development of drugs targeting downstream players in the PD-1-dependent signaling pathway has been hampered by our poor understanding of the molecular details of the intermolecular interactions involved in the pathway. Activation of PD-1 leads to phosphorylation of two signaling motifs located in its cytoplasmic domain, the immune tyrosine inhibitory motif (ITIM) and immune tyrosine switch motif (ITSM), which recruit and activate protein tyrosine phosphatase SHP2. This interaction is mediated by the two Src homology 2 (SH2) domains of SHP2, termed N-SH2 and C-SH2, which recognize phosphotyrosines pY223 and pY248 of ITIM and ITSM, respectively. SHP2 then propagates the inhibitory signal, ultimately leading to suppression of T cell functionality. In order to facilitate mechanistic structural studies of this signaling pathway, we report the resonance assignments of the complexes formed by the signaling motifs of PD-1 and the SH2 domains of SHP2.

## Biological context

The recent success of anticancer therapies that target immune checkpoint receptor PD-1 has sparked considerable interest in the molecular details behind its signaling function. PD-1 is a 288-amino-acid receptor of the CD28 family, mostly expressed on the surface of T lymphocytes, whose main function is to maintain immune tolerance and prevent overactive T cell responses (Boussiotis 2016). However, PD-1 functionalities are also exploited by certain cancer types to evade immune surveillance; for this reason, monoclonal antibodies that block the interaction between this receptor and its activation ligand PD-L1 have proven successful in the treatment of tumors such as metastatic melanoma, non-small cell lung cancer and renal cell carcinoma (Page et al. 2014; Topalian et al. 2015). Despite their efficacy, the very high cost of immunotherapies poses a severe burden on public healthcare systems and calls for novel, equally effective, but more affordable drugs (Prasad et al. 2017).

Targeting the PD-1-dependent signaling pathway has been made problematic by our limited knowledge of the molecular events following PD-1 activation; only recently have ground-breaking studies elucidated important mechanistic aspects of the PD-1-dependent signaling events. Activation of PD-1 leads to phosphorylation of two key tyrosine residues in its cytoplasmic domain by Src family kinases (Sharpe and Pauken 2018; Hui et al. 2017). These two tyrosines, Y223 and Y248, are embedded into two immune-tyrosine signaling motifs, which are unique among the members of the CD28 family, namely the immune-tyrosine inhibitory motif (ITIM) and immune-tyrosine switch motif (ITSM), respectively (Riley 2009). The two phosphotyrosines recruit and activate Src homology 2 (SH2) domain-containing phosphatase 2 (SHP2), which propagates the signal from PD-1 by dephosphorylating key tyrosines on CD28 (Hui et al. 2017).

SHP2 is a 70-kDa protein of the PTP (protein tyrosine phosphatase) superfamily, which consists of three folded domains (two SH2 domains arranged in tandem, termed N-SH2 and C-SH2, and a catalytic PTP domain) followed by a disordered C-terminal tail with putative regulatory functions (Neel et al. 2003). In its basal state, SHP2 activity is very low, due to an auto-inhibitory interaction between the N-SH2 and the PTP, which occludes the catalytic site and prevents substrate processing (Hof et al. 1998). Engagement of the N-SH2 or both the N-SH2 and the C-SH2 by monovalent or divalent phosphopeptides, respectively, leads to the transition to an open conformation, in which the N-SH2 is displaced and the catalytic site becomes accessible (Barford and Neel 1998). Mutations that disrupt the interaction between the N-SH2 and the PTP also favor the open conformation and have been associated with several diseases (Keilhack et al. 2005).

SH2 domains are protein modules of around 100 amino acids, with a conserved fold consisting of a central three-stranded antiparallel β sheet flanked by two α helices, whose function is to recognize phosphotyrosine-containing peptides (Waksman et al. 2004). In this work, the nomenclature for SH2–phosphopeptide complexes follows the convention introduced by Eck and coworkers (Eck et al. 1993): the SH2 helices are named αA and αB, the β strands are βA–βG and the loops are defined based on the structural elements that they connect; phosphopeptide residues are numbered according to their position relative to the phosphotyrosine (pY–2, pY–1, pY+1, pY+2…).

The structural and biochemical details of the activation of SHP2 by PD-1 derived phosphopeptides have been reported recently (Hui et al. 2017; Peled et al. 2018; Marasco et al. 2020). Interestingly, a peptide containing both ITIM and ITSM linked together is required for maximal stimulation of phosphatase activity. This bidentate peptide binds the N-SH2 and C-SH2 domains of one SHP2 molecule with ITIM and ITSM, respectively, to form a 1:1 doubly bound heterodimeric complex. This dual binding event requires a large rearrangement of the orientation of the SH2 domains in SHP2 in order to satisfy the spatial restraints imposed by the linker between ITIM and ITSM. Therefore, formation of the 1:1 doubly bound complex is associated with overcoming a high conformational energy barrier, which slows down the association of the second SH2 domain with the second pY motif of the same peptide both in vitro and in vivo (Marasco et al. 2020; Oh et al. 2012). Consequently, the stoichiometry and oligomeric state of the complexes present in a mixture of SHP2 and bidentate peptide is heterogeneous, particularly in regimes of high protein concentrations or receptor clustering, with variable amounts of higher-order oligomeric protein–peptide complexes (wherein the SH2 domains of a single protein molecule engage pY motifs of different peptide molecules) in addition to the 1:1 doubly-bound heterodimer (Marasco et al. 2020; Oh et al. 2012).

Here, we report the backbone resonance assignments for the unbound, ITIM-bound and ITSM-bound states of the N-SH2 and C-SH2 domains. In addition, we report the assignment of the protein side-chains and bound peptide resonances of the N-SH2–ITIM complex; the corresponding resonance assignment for the C-SH2–ITSM complex has been published previously (Marasco et al. 2020). These results have aided the assignment of the backbone resonances of tSH2 (a construct containing both N-SH2 and C-SH2 domains) in complex with the bidentate peptide ITIM-[dPEG4]_2_-ITSM, which contains both ITIM and ITSM joined by a polyethyleneglycol-based linker. The assignment of the resonances of the tSH2 and their shifts upon titration with the bidentate peptide revealed the presence of a heterogeneous mixture of 1:1 heterodimers and higher-order protein–peptide oligomers at variable stoichiometric ratios (Marasco et al. 2020).

## Methods and experiments

### Protein expression and purification

The DNA sequences of human N-SH2 (SHP2^1−105^), C-SH2 (SHP2^106−220^) and tSH2 (SHP2^1−220^) were cloned into the pETM22 expression vector, which allows for expression of the target proteins as fusion constructs with a cleavable His_6_-thioredoxin tag. The vectors were transformed into Tuner (DE3) competent cells (Merck). For recombinant protein expression, freshly transformed cells were grown at 37 °C to an optical density (OD) of 0.6–0.8. Afterwards, the culture was quickly chilled and 0.1 mM of isopropyl β-d-1-thiogalactopyranoside (IPTG) was added to induce protein expression, which was continued for 20 h at 20 °C. Preparation of U-^13^C,^15^N samples (N-SH2, C-SH2 and tSH2) was achieved by growing the bacteria in minimal medium containing ^15^NH_4_Cl (1 g/l, Cambridge Isotope Laboratories) and ^13^C-D-glucose (4 g/l, Cambridge Isotope Laboratories) as the sole nitrogen and carbon sources, respectively. Due to its larger size and tendency to form oligomers, tSH2 in complex with the bidentate ITIM-[dPEG4]_2_-ITSM peptide required sparse deuteration, which was achieved by growing the cells in deuterated minimal medium with protonated carbon source.

After protein expression, the cultures were harvested, pelleted and stored at − 20 °C until further use. Cells were lysed by sonication in wash buffer (1 M NaCl, 50 mM Tris–HCl, 5% glycerol, 10 mM imidazole, 5 mM β-mercaptoethanol, pH 7.6) supplemented with one tablet of EDTA-free cOmplete™ protease inhibitors (Roche), 100 µg of lysozyme (Roth) and 50 µg of DNAse (NEB). The lysate was clarified by centrifugation at 18000×*g* for 1 h and the filtered supernatant was loaded on a HisTrap HP column (GE Healthcare), previously equilibrated with wash buffer. After loading, the column was washed extensively (10 column volumes, CV) with wash buffer, before elution of the bound protein with 5 CV of elution buffer (1 M NaCl, 50 mM Tris–HCl, 5% glycerol, 500 mM imidazole, 5 mM β-mercaptoethanol, pH 7.6). The fractions containing the protein were pooled and 3C protease (1:100 protease:protein ratio) was added to cleave the His_6_-thioredoxin tag. Excess imidazole was removed by dialyzing the eluate against 2 l of wash buffer at 4 °C overnight. Purification continued the following day with a second round of affinity chromatography (HisTrap) to separate the His_6_-thioredoxin tag from the target protein. The fractions containing the protein of interest were concentrated to a final volume of 1−2 ml and loaded on a HiLoad 16/600 Superdex 75 pg column (GE Healthcare), previously equilibrated with NMR buffer (100 mM MES, 150 mM NaCl, 3 mM TCEP, 0.05% NaN_3_, pH 6.8). The fractions containing pure protein were pooled, the protein concentrated to the desired concentration and either used directly for experiments or flash-frozen with liquid nitrogen for long-term storage at − 80 °C. Sample purity was confirmed by SDS-PAGE.

PD-1 immune tyrosine motifs were purchased as synthetic phosphopeptides (ITIM: Ac-FSVDpYGELDFQ-NH_2_; ITSM: Ac-EQTEpYATIVFP-NH_2_) from Caslo ApS (Lyngby, Denmark). The bidentate peptide ITIM-[dPEG4]_2_-ITSM was made by connecting ITIM and ITSM with two discrete poly-(ethylene glycol)-4 units, in order to match the length of the linker that separates ITIM and ITSM in wild-type PD-1, and was purchased from the same company (Sequence: Ac-FSVDpYGELDFQ-[dPEG_4_]-[dPEG_4_]- EQTEpYATIVFP-NH_2_).

### NMR spectroscopy

NMR assignment spectra were recorded at a temperature of 298 K on Bruker Avance III HD 600 MHz and 850 MHz spectrometers running Topspin 3.2 software and equipped with N_2_-cooled and He-cooled inverse HCN triple-resonance cryogenic probeheads, respectively. Protein concentrations ranged from 500 µM to 800 µM; for peptide-containing samples, peptides were added in two-fold excess unless specified otherwise below. For tSH2 in complex with ITIM-[dPEG4]_2_-ITSM, the peptide was in 1.5-fold excess with respect to the protein.

Backbone resonances of N-SH2, C-SH2 and their ITIM- and ITSM-bound forms, and those of unbound tSH2, were assigned using the standard suite of triple-resonance experiments (2D ^15^N-HSQC, 3D HNCO, 3D HNCACB and 3D HN(CO)CACB) **(**Kay et al. 1994; Muhandiram and Kay 1994; Yamazaki et al. 1994) and the sidechain resonances of N-SH2–ITIM were assigned from 3D HC(C)H-TOCSY (Kay et al. 1993), 3D H(CCCO)NH-TOCSY (Logan et al. 1992), 3D NOESY-^15^N-HSQC (Marion et al. 1989), 2D HBCB(CGCD)HD, 2D HBCB(CGCDCE)HE (Yamazaki et al. 1993) and 2D constant-time ^13^C-HSQC spectra (Vuister and Bax 1992). Assignments of proton resonances of ITIM in complex with N-SH2 were obtained from 2D ^13^C,^15^N-filtered NOESY and 2D ^13^C,^15^N-filtered TOCSY spectra (Zwahlen et al. 1997) recorded on a sample in which the protein was in excess with respect to the peptide; deuterated PIPES instead of unlabeled MES was used as a buffer in order to minimize the *t*_1_-noise from buffer peaks. For tSH2 in complex with ITIM-[dPEG4]_2_-ITSM, backbone resonance assignment was achieved by TROSY-based triple-resonance experiments (2D TROSY-HSQC, 3D TROSY-HNCO, 3D TROSY-HNCACB and 3D TROSY-HN(CO)CACB) (Pervushin et al. 1997; Salzmann et al. 1998) and by chemical-shift comparison with previously assigned N-SH2 and C-SH2 complexes. All the spectra were processed with Topspin 3.2 (Bruker) or NMRpipe (Delaglio et al. 1995). Peak picking and resonance assignment were done with CcpNmr Analysis (Vranken et al. 2005).

### Assignments and data deposition

**Fig. 1:**
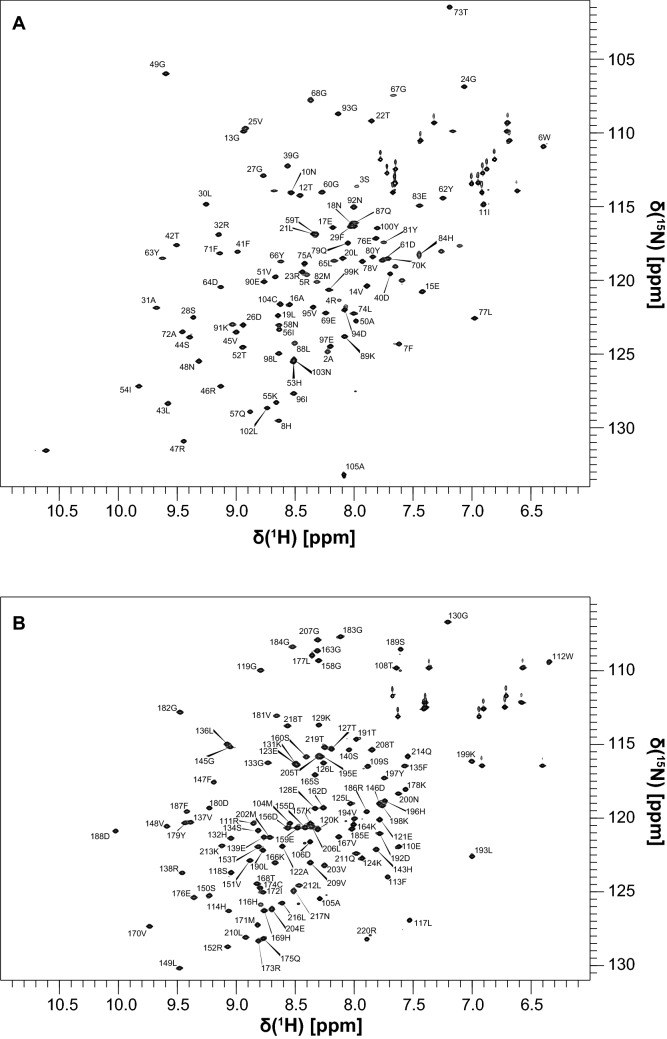
2D ^1^H,^15^N HSQC spectra of N-SH2 (**a**) and C-SH2 (**b**) with the corresponding assignments. The spectra were collected on a 600 MHz Bruker Avance III spectrometer

The features of the ^15^N HSQC spectra of N-SH2 and C-SH2 confirmed that these two domains adopt a stable fold in solution (Fig. 1). Excluding prolines and the N-terminal residues generated by 3C protease cleavage (whose signals were missing in the spectra, probably due to rapid solvent exchange), the backbone assignments of N-SH2 and C-SH2 were 93% and 96.4% complete, respectively. For N-SH2, the missing signals belonged to the segment R32–G39 (BC loop), which is part of the phosphotyrosine binding region. This segment is known to be poorly structured in SH2 domains in the absence of bound phosphopeptides (Booker et al. 1992). Similarly, the amide peaks of Q140 and S141 of C-SH2 were missing. Furthermore, the amide peaks of residues H85 and G86 of N-SH2, which join the BG loop to αB, were also missing.

**Fig. 2:**
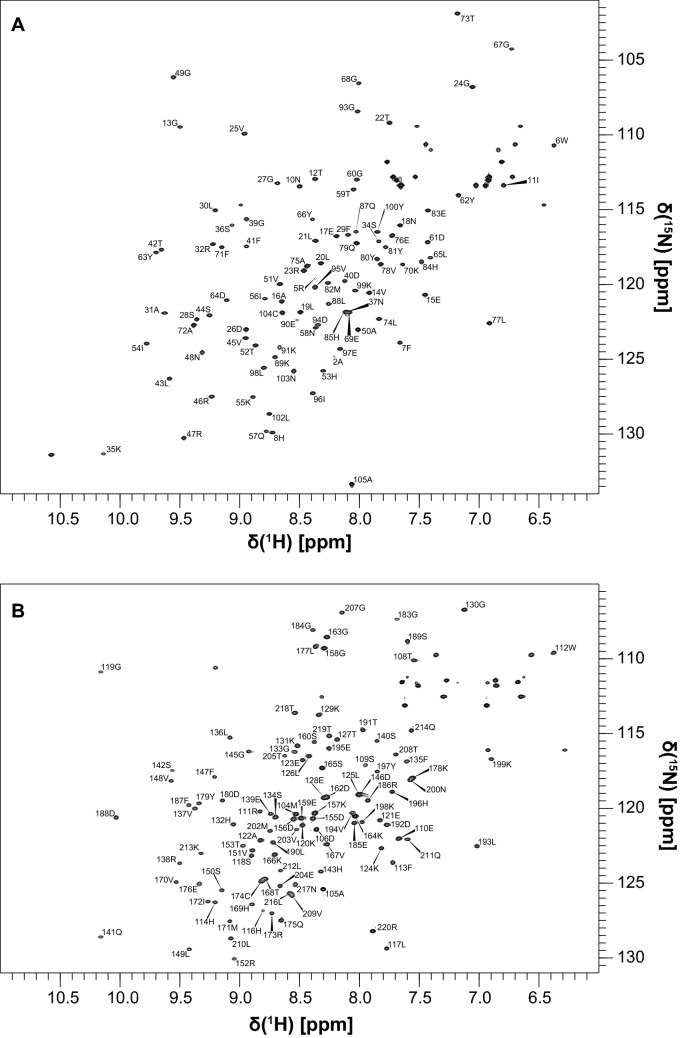
2D ^1^H,^15^N HSQC spectra of N-SH2–ITIM (**a**) and C-SH2–ITIM (**b**) with the corresponding assignments. The spectra were collected on a 600 MHz Bruker Avance III spectrometer

**Fig. 3:**
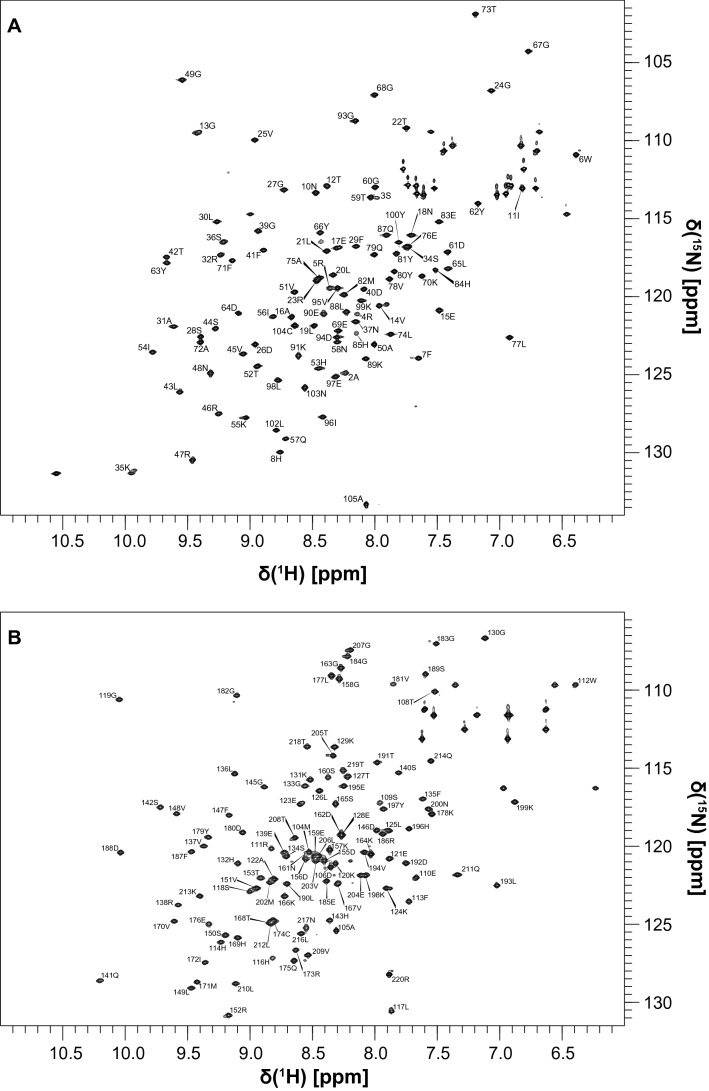
2D ^1^H,^15^N HSQC spectra of N-SH2–ITSM (**a**) and C-SH2–ITSM (**b**) with the corresponding assignments. The spectra were collected on a 600 MHz Bruker Avance III spectrometer

As expected, addition of PD-1-derived phosphopeptides led to the structuring of the pY-binding region: the assignments of N-SH2–ITIM and N-SH2–ITSM were complete except for G86 and N92, while the assignment of C-SH2–ITSM was 100% complete. On the other hand, in the C-SH2–ITIM complex several amide peaks were missing (G115, G154, N161, V181, G182 and L206) (Figs. 2 and 3). In general, the chemical shifts perturbations induced by ITIM and ITSM on the SH2 domains of SHP2 are different.

**Fig. 4:**
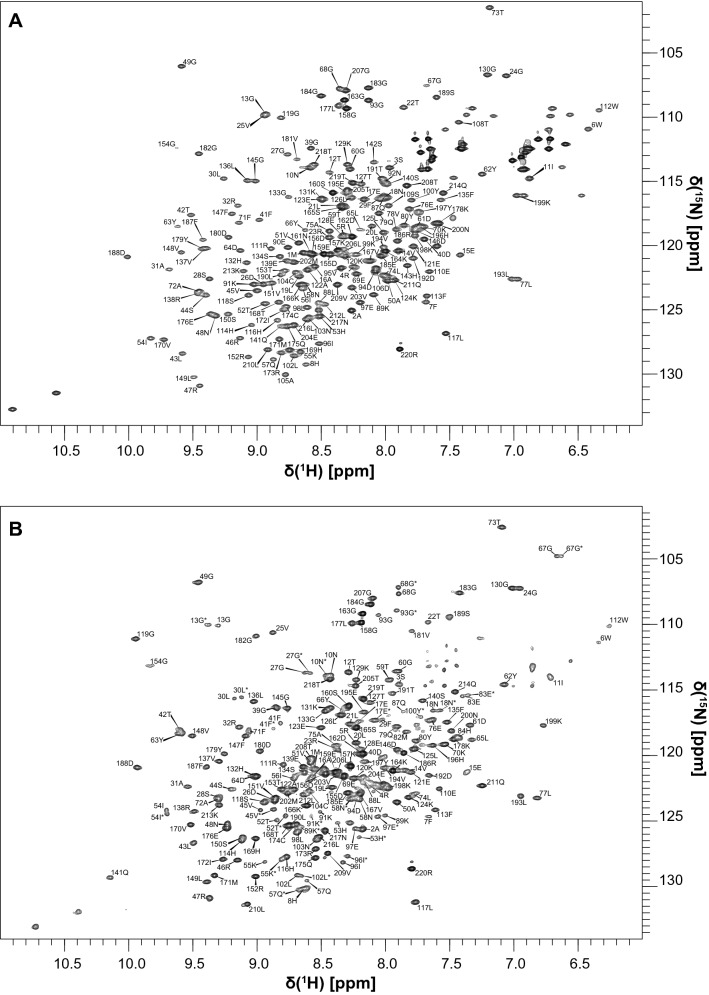
2D ^1^H,^15^N HSQC spectrum of tSH2 (**a**) and 2D ^1^H,^15^N TROSY HSQC spectrum of tSH2–ITIM-[dPEG4]_2_-ITSM (**b**) with the corresponding assignments. In the latter, the resonances belonging to the state in which N-SH2 is bound to ITIM are marked with an asterisk. The spectra were collected on a 850 MHz Bruker Avance III spectrometer

**Fig. 5:**
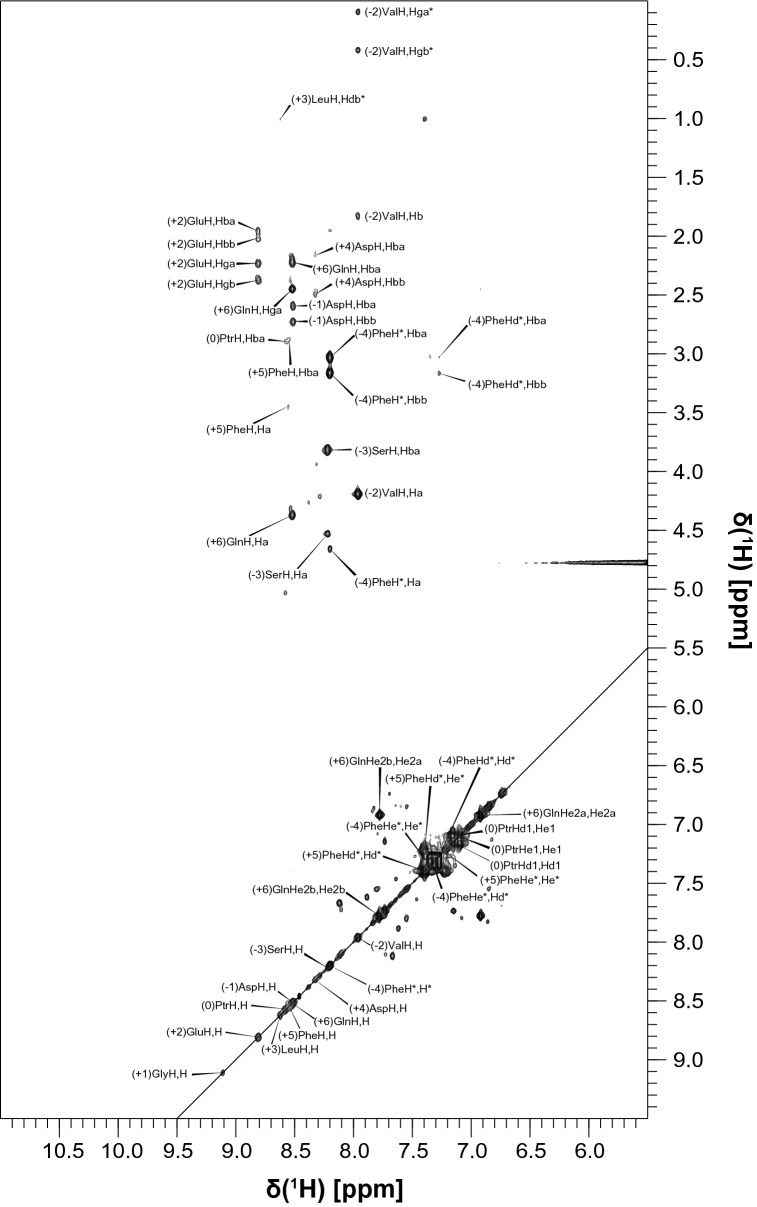
Excerpt of the ^13^C,^15^N-filtered TOCSY spectrum of N-SH2–ITIM, which shows the assignment of the amide region of ITIM. H* indicates the N-terminal amide hydrogen. The spectra were collected on a 600 MHz Bruker Avance III spectrometer

The ^15^N HSQC spectrum of unbound tSH2 closely resembles the overlay of the spectra of unbound N-SH2 and C-SH2, which made the assignment relatively simple by comparison of the chemical shifts. The assignments could be transferred from the isolated SH2 domains to the tSH2 construct, except for the BC loop of N–SH2 (S34–N37) and the Y81–H85 segment (Fig. 4a). The bound state of tSH2 was measured in the presence of a 1.5-fold excess of biphosphorylated ITIM-[dPEG4]_2_-ITSM and a tSH2 concentration of 500 µM; the ^15^N TROSY spectrum at this stoichiometric ratio and protein concentration reveals the presence of two distinct peaks for several amide groups (Fig. 4b). By comparison of this spectrum with those of N-SH2–ITIM, N-SH2–ITSM, C-SH2–ITIM and C-SH2–ITSM, it was possible to establish that the two resonances represent two different states of tSH2, one in which N-SH2 is bound to ITIM and one in which N-SH2 is bound to ITSM. The C-SH2 appears to be bound only to ITSM. This indicates that binding of ITIM-[dPEG4]_2_-ITSM to tSH2 results in a heterogeneous mixture of complexes of different architecture. Contrary to the bound states of isolated N-SH2, the peaks of the BC loop were still missing in this complex. In addition, the segment A105–T108, corresponding to the linker between N-SH2 and C-SH2, did not yield any observable amide peaks, presumably due to either an unfavorable conformational exchange regime or rapid proton-exchange with the solvent. Due to its physiological importance (ITIM is the specific binder of N-SH2 and is directly responsible for maximum activation of SHP2), the assignment of the resonances of the N-SH2-ITIM complex also included protein side-chains and peptide proton resonances (Fig. 5).

The ^1^H, ^13^C, ^15^N backbone (^1^HN, ^15^N, ^13^Cα, ^13^Cβ, ^13^CO) resonance assignments of N-SH2, C-SH2, tSH2, C-SH2–ITIM and N-SH2–ITSM have been deposited at the BioMagResBank (https://www.bmrb.wisc.edu) under accession codes 28069, 28070, 28071, 28072 and 28073, respectively. The backbone and sidechain resonances of N-SH2–ITIM are available under accession code 28074, while those of C-SH2–ITSM have been published as part of previous work (BMRB code 34384) (Marasco et al. 2020). Due to the heterogeneous population of bound states, the backbone chemical shifts of the tSH2–ITIM-[dPEG4]_2_-ITSM have been deposited as two separate groups: those corresponding to the state in which N-SH2 is bound to ITIM (BMRB code 28075) and those corresponding to the state in which N-SH2 is bound to ITSM (BMRB 28076).
